# The *MHC Class Ia* Genes in Chenfu’s Treefrog (*Zhangixalus chenfui*) Evolved via Gene Duplication, Recombination, and Selection

**DOI:** 10.3390/ani10010034

**Published:** 2019-12-23

**Authors:** Hu Chen, Siqi Huang, Ye Jiang, Fuyao Han, Qingyong Ni, Yongfang Yao, Huailiang Xu, Sudhanshu Mishra, Mingwang Zhang

**Affiliations:** College of Animal Science and Technology, Sichuan Agricultural University, 211# Huimin Road, Chengdu 611130, China; aquaticpractitioner@stu.sicau.edu.cn (H.C.); seven17huang@dingtalk.com (S.H.); jiangye20170907@163.com (Y.J.); hanfuyao@126.com (F.H.); niqingyong@hotmail.com (Q.N.); yaoyongf@126.com (Y.Y.); huailxu@yahoo.com (H.X.); mishra.sudhanshu30@gmail.com (S.M.)

**Keywords:** amphibian, paralog, immunogenetics, polymorphism, molecular evolution

## Abstract

**Simple Summary:**

Amphibians, the first terrestrial vertebrates, provide materials for adaptive evolutionary studies, such as the evolution of the major histocompatibility complex (MHC). To date, various MHC evolutionary mechanisms have been identified in frogs, but more research is needed to determine the evolutionary mechanisms of the frog MHC. The main purpose of this study was to evaluate polymorphisms in the *MHC class Ia* genes of the Chenfu’s Treefrog. The *MHC class Ia* genes of the Chenfu’s Treefrog have high polymorphism. The mechanisms responsible for the formation of the polymorphisms include gene duplication, recombination, and selection.

**Abstract:**

The molecular mechanisms underlying the evolution of adaptive immunity-related proteins can be deduced by a thorough examination of the major histocompatibility complex (MHC). Currently, in vertebrates, there is a relatively large amount of research on MHCs in mammals and birds. However, research related to amphibian *MHC* genes and knowledge about the evolutionary patterns is limited. This study aimed to isolate the *MHC class I* genes from Chenfu’s Treefrog (*Zhangixalus chenfui)* and reveal the underlying evolutionary processes. A total of 23 alleles spanning the coding region of *MHC class Ia* genes were identified in 13 individual samples. Multiple approaches were used to test and identify recombination from the 23 alleles. Amphibian *MHC class Ia* alleles, from NCBI, were used to construct the phylogenetic relationships in MEGA. Additionally, the partition strategy was adopted to construct phylogenetic relationships using MrBayes and MEGA. The sites of positive selection were identified by FEL, PAML, and MEME. In Chenfu’s Treefrog, we found that: (1) recombination usually takes place between whole exons of *MHC class Ia* genes; (2) there are at least 3 loci for *MHC class Ia*, and (3) the diversity of genes in *MHC class Ia* can be attributed to recombination, gene duplication, and positive selection. We characterized the evolutionary mechanisms underlying *MHC class Ia* genes in Chenfu’s Treefrog, and in so doing, broadened the knowledge of amphibian MHC systems.

## 1. Introduction

Genes of the major histocompatibility complex (MHC), as cell surface glycoproteins, are vital for adaptive immunoreactions. To distinguish between intracellular and extracellular antigens, there are two main subgroups of MHC: class I and class II. A molecule of *MHC class I* consists of a microglobulin chain (β_2_ m chain) and a heavy chain (α chain). This molecule connects peptides from intracellular pathogens and displays it at the surface of the cell where an immunoreaction is initiated upon recognition by CD8^+^ T cells [1,2,3]. The heavy chain that is encoded by classical *MHC class I* genes has a cytoplasmic region, a transmembrane region, and three extracellular domains designated α1, α2, and α3. There are seven exons in the *MHC class I* genes. Specifically, the leader peptide is encoded by exon 1; three extracellular domains are encoded by exons 2, 3, and 4 (α1, α2, and α3, respectively); the transmembrane domain is encoded by exon 5; and the cytoplasmic tail is encoded by exons 6 and 7. The antigen-binding sites (ABS) are encoded by the second and third exons in the α1 and α2 domains, respectively [1,4]. Therefore, a high degree of genetic diversity exists in the second and third exons of the classical genes of *MHC class I* [5], and MHC genes have been the subject of evolutionary ecology studies in a wide variety of species over the last two decades [6].

In terms of research on the MHC, a lot of attention has been given to the mechanisms by which evolution can favor diversity in *MHC class I* genes. There are three mechanisms of particular interest, recombination [7,8,9,10], positive selection [11,12,13,14], and gene duplication [15,16]. Under the action of these three mechanisms, a high degree of gene polymorphism [17,18,19] and increased variation in loci numbers [15,20,21] exist in *MHC class I* genes. The high number of genetic polymorphisms of MHC genes is thought to offer organisms protection against a large spectrum of pathogens and therefore increases their fitness. This hypothesis is supported by the associations observed between disease susceptibility and MHC gene diversity in numerous vertebrates [22,23,24].

The evolution of *MHC class I* genes have mainly been studied in fish, mammals, and birds [6,10,25,26]. The relatively under studied group, amphibians, should be given special attention, not only because they are the first terrestrial vertebrates but because their unique living environment exposes them to a very different spectrum and diversity of microbes. Nonetheless, relatively few studies have explored amphibian *MHC class I* genes. The existing evolutionary studies on amphibian *MHC class I* genes have been reported in Urodela and Anura [27,28,29,30,31]. These studies have revealed that the evolution of *MHC class I* gene can be credited to recombination and positive selection [28,29]. Moreover, amphibian *MHC class I* genes commonly contain trans-species polymorphisms [29]. However, the evolutionary mechanisms responsible for amphibian *MHC class Ia* genes polymorphisms may be more complicated than previously thought [31,32]. Consequently, to gain a more systematic understanding of the evolution of *MHC class I* genes, more amphibians should be examined. Chenfu’s Treefrog is a species of frog in the family Rhacophoridae endemic to China where it is found in Sichuan, Guizhou, Hubei, and Fujian provinces [33]. Its natural habitats are temperate forests, subtropical moist lowland forests, subtropical moist montane forests, subtropical moist shrubland, freshwater marshes, rural gardens, ponds, and irrigated land. The aim of this study was to identify *MHC class I* genes in the Chenfu’s Treefrog. In addition, we investigated whether selective mechanisms and gene replication affected MHC mutations. This will help to further understand the evolution of the amphibian *MHC class I*.

## 2. Materials and Methods

### 2.1. Sampling and RNA Isolation

Thirteen healthy Chenfu’s Treefrogs were collected from the Wawu Shan Nature Reserve in Sichuan, China. The experimental frogs were euthanized with 95% alcohol. The liver was dissected and frozen in liquid nitrogen, before freezing at −80 °C for storage until RNA extraction. Trizol^®^ reagent was used to isolate the total RNA from each sample according to the manufacturer’s protocol (TaKaRa, Otsu, Japan). At the wavelengths of 280 nm and 260 nm, the optical density absorption ratio (Bio-Rad) was measured to determine the RNA concentrations. Specimens with the rate of absorption from 1.7 to 2.2 were used for cDNA synthesis. RNA integrity was measured using gel electrophoresis. Sichuan Agricultural University’s Animal Ethics Committee approved all experiments with live tree frogs with approval number, 71234-2015-0901.

### 2.2. cDNA Amplification and Cloning

Prime Script RT Reagent Kit (TaKara, Dalian, China) was used for 1st strand cDNA synthesis, which contained mixture of equal amounts of liver RNA (2 µg) from the 13 individuals. We first amplified a partial Chenfu’s Tree frog MHC (ZcMHC) sequence with primers ZcMHC-F and ZcMHC-R (Table 1). PCR condition was a touchdown PCR (2× Taq PCR Mastermix, Tiangen, Beijing, China): initial denaturation at 94 °C for 5 min; 10 cycles for 30 s at 94 °C, 68 °C for 30 s (−1 °C per cycle), and 72 °C for 2 min; 25 cycles for 30 s at 94 °C, 58 °C for 30 s, and 72 °C for 2 min; final extension at 72 °C for 10 min; and cooled to 4 °C. PCR products were excised from agarose gel (Universal DNA Purification Kit, TIANGEN, China), cloned into pMD19-T (TaKaRa, Dalian, China), and 30 clones were sequenced (BBI Life Science Corporation Company, Shanghai, China).

We then obtained full-length cDNA sequences of *MHC class Ia* by 5’ and 3’ RACE PCR (SMART RACE cDNA Amplification Kit, Clontech, CA, USA), with primers ZcMHC-F1, ZcMHC-F2, ZcMHC-R1, and ZcMHC-R2 (Table 1) designed based on the partial sequence, from the mixed RNA as above. The PCR condition was the touchdown PCR as above. PCR products were excised from agarose gel, cloned into pMD19-T, and 30 clones were sequenced as above.

In order to obtain individual allelic sequences, individual liver RNAs (2 µg each) were separately subjected to cDNA synthesis (Prime Script RT Reagent Kit, TaKaRa, Dalian, China) and touchdown PCR of coding sequences with primers ZcMHC-F3 and ZcMHC-R3 (Table 1) designed based on the full-length cDNA sequence. PCR products were cloned into pEASY-T5 Zero vector (TransGen Biotech, Beijing, China). We picked 30 clones up of each PCR product and sequenced as above.

### 2.3. Analysis of ZcMHC Class Ia

Sequence assembly was performed by DNASTAR version 7.1 (DNASTAR, Inc, Madison WI, USA). Using BLASTp and BLASTn (http://www.ncbi.nlm.nih.gov), the deduced protein sequences and nucleotides were studied. In addition, the ORF was predicted using the Open Reading Frame Finder (http://www.ncbi.nlm.nih.gov/gorf/gorf.html). With the use of NetOGlyc 3.1 Server (http://www.cbs.dtu.dk/services/NetOGlyc-3.1/) and NetNGlyc 1.0 Server (http://www.cbs.dtu.dk/services/NetNGlyc), posttranslational modification forecasts of N- and O-glycosylation sites were determined. Furthermore, transmembrane domain topology for proteins was predicted using the TMHMM Server v2.0 (http://www.cbs.dtu.dk/services/TMHMM/).

### 2.4. Sequence Alignment and Allele Identification

In order to accurately obtain the full-length ORF fragments (about 1100 bp), we used forward and reverse primers to obtain the sequence from both ends for each clone. Two ABI trace files from each clone were examined, edited, and assembled using DNASTAR 7.1. ClustalW in MEGA5 was used to align the assembled sequences. Only the sequences that appeared more than twice were considered alleles and included.

Newly identified alleles were translated into amino acid sequences and aligned with human HLA [5]. The identification of major amino acid sites in disulfide bond formation and peptide binding was permitted in the amino acid sequences.

### 2.5. Statistical Study

#### 2.5.1. Recombination Detection

MEGA5 (https://www.megasoftware.net/ was used to compute the distances of Poisson-corrected amino acid and the average pairwise nucleotide distances (Kimura-2-parameter model, K2P). A total of 1000 bootstrap replicates were used to obtain standard errors of the estimates. A number of approaches were used to detect the recombination in our data: BOOTSCAN, RDP, CHIMAERA, MAXCHI, GENECONV, 3SEQ, and SISCAN, which were all implemented in the RDP4 (namely Recombination Detection Program version 4). The highest acceptable *p* value for events of recombination was set at 0.01 with a window size of 20 nucleotides in order to minimize the false-positive error rate. Only the breakpoints, identified by more than four approaches were considered valid.

#### 2.5.2. Phylogenetic Analyses

The amphibian *MHC class Ia* alleles in the NCBI database (https://www.ncbi.nlm.nih.gov/) were used to construct phylogenetic relationships. *MHC class Ib* genes from *Xenopus laevis* and *Xenopus tropicalis* and *MHC class Ia* genes from *Polypedates megacephalus*, *Lithobates catesbeianus*, *Espadarana prosoblepon*, *Ambystoma mexicanum*, *Xenopus ruwenzoriensis*, *Zhangixalus omeimontis*, *Rana huanrenensis, Agalychnis callidryas*, *Smilisca phaeota*, and *Xenopus laevis*, were included in the phylogenetic analysis. MEGA5 was utilized to construct a neighbor-joining (NJ) tree in order to determine the general phylogenetic state of the genes of *MHC class I*, which were newly isolated. Later, according to the results of recombination tests, the sequences were partitioned into individual regions to deduce more precisely the phylogenetic trees of anuran *MHC class I* genes, given that the identification of true phylogenetic relationship can be hindered by events of recombination. For inclusion in the tree construction, around three alleles from each species with *MHC class I* gene sequences from the NCBI database were chosen as representatives. Moreover, according to the Akaike Information Criterion (AIC) in MrModeltest, the most suitable model of nucleotide substitution was selected. Analyses revealed that GTR+I was the most appropriate model for all domains (α1, α2, and α3). MrBayes 3.2 (http://nbisweden.github.io/MrBayes/index.html) was used to reconstruct the Bayesian inference trees. Until the standard deviation of the split frequencies became <0.01, two independent runs of four Metropolis with simulations of MCMC (namely Markov Chain Monte Carlo) (three of them were ‘heated’ at the temperature of 0.1) were conducted for millions of generations. From the cold chain, the first quarter of the sampled trees were abandoned by default. The rest of the trees were used to calculate the posterior probability of every bipartition and the major rule consensus tree. In addition, the matrix of nucleotide distances was utilized via MEGA5 to construct the neighbor-joining trees of individual exons. A total of 1000 bootstrap replicates were used to estimate support for the nodes in the obtained tree.

#### 2.5.3. Positive Selection Identification

False inferences of positive selection were ascribed to recombination. However, according to breakpoints of recombination which had been deduced, false positive can be decreased to an acceptable degree with the partitioning strategy in consideration of separate un-recombinant segments [34]. With the purpose of testing whether positive selection could shape the evolutionary pattern of the sequences of *MHC class I* isolated in this study, three methods were utilized for pre-analysis partitioning. Firstly, the Codeml subroutine in PAML version 4 (http://abacus.gene.ucl.ac.uk/software/paml.html) was used to test for the existence of positive selection signals. This procedure could be used to generate the maximum possibility forecasts of ω (ω = dN/dS, which means the synonymous/non-synonymous substitution ratios) amongst codons. Corresponding to the different distributing patterns of ω, six different methods could be used for verification in our statistics, including M8 (positive selection with the beta distribution approximating ω variation), M7 (almost neutral with the beta distribution approximating ω variation), M3 (discrete), M2a (positive selection), M1a (almost neutral), and M0 (one ω). In order to test the existence of positive selection in M2a, M3, and M8 models, three likelihood ratio tests (LRTs) were conducted for comparison of the nested models (M7 vs. M8, M1a vs. M2a, and M0 vs.M3). In models M2a and M8, Bayes empirical Bayes (BEB) method was used to identify positively selected sites. However, provided that the Codeml power would be influenced by the preciseness of the inferred phylogenetic trees, the two other approaches to detect the signals of selection were also utilized. Those two approaches, MEME, and FEL, were conducted at the website Data monkey (http://www.datamonkey.org). The combined results from all available studies were used to obtain a more precise understanding of the sites under positive selection.

## 3. Results

### 3.1. Identification and Sequencing of ZcMHC Class Ia cDNA

**Figure 1:**
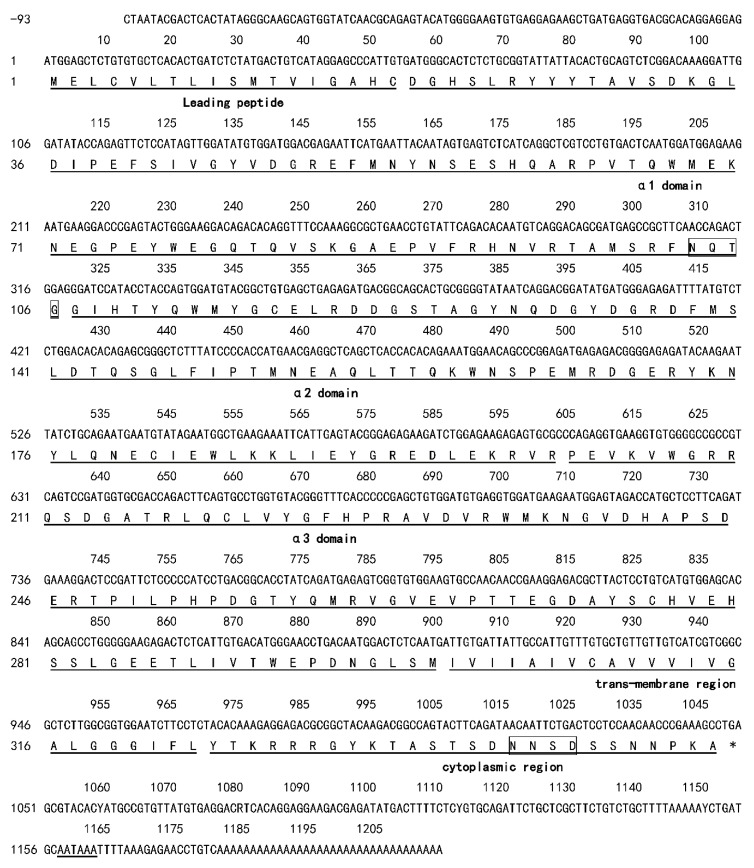
Nucleotide and predicted amino acid sequences of ZcMHC class Ia. The regions are marked by underline and the region name. The stop codon is indicated by an asterisk. N-glycosylation sites are marked with squares. The polyadenylation signal (AATAAA) is underlined in the 3′ UTR.

As shown in Figure 1, GenBank (https://www.ncbi.nlm.nih.gov/) accession number: KX021343, Zc*MHC class Ia* cDNA was obtained from the liver by RACE PCR and RT-PCR. A 1050 bp open reading frame (namely ORF), a 93 bp 5′-untranslated region, and a 165 bp -untranslated region were contained in the 1308 bp full-length Zc*MHC class Ia* cDNA. The 3′UTR contained a consensus polyadenylation signal sequence (AATAAA), which was 18 bp upstream from the poly A tail. The ORF encoded a 349-amino-acid precursor consisting of an α1 domain of 88 amino acids, a putative leader peptide of 18 amino acids, an α2 domain of 93 amino acids, and an α3 domain of 101 amino acids, followed by a 26 amino acid cytoplasmic region, and a 23 amino acid transmembrane region. According to the analyses of potential N-linked glycosylation sites, the α1 domain and cytoplasmic region contained potential N-linked glycosylation sites with amino acids NQTG and NNSD. Moreover, a potential O-linked glycosylation site was found at amino acid site 257.

### 3.2. Allele Characterization of ZcMHC Class Ia

**Figure 2:**
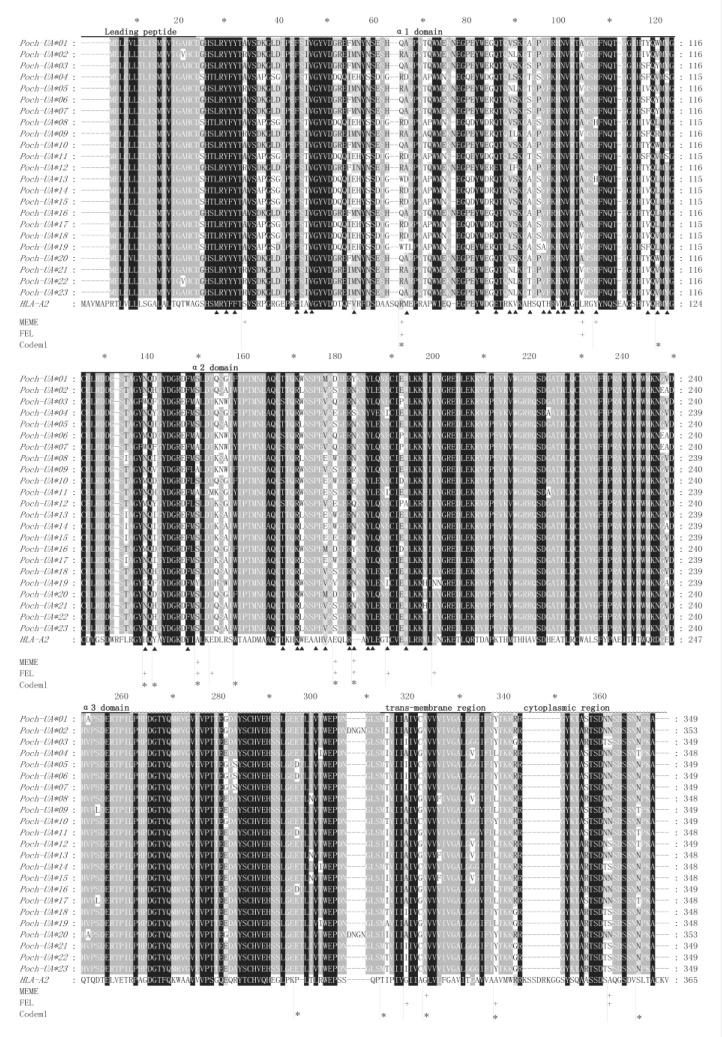
Alignments of amino acid sequences translated from the 23 *MHC class I* alleles. Sites marked by black triangle represent the putative ABS deduced from structural information from human HLA molecules. The predicted positive selection sites were marked by the short vertical line. * refer to amino acids predicted to be under positive selection in the M8 model of PAML, with posterior probabilities of >95%. + marks positively selected sites identified by FEL and MEME.

We obtained 390 sequences using the ZcMHC-F3 and ZcMHC-R3 primers (Table 1) and identified 26 putative alleles of *MHC class I*. They contained the complete coding regions with lengths of 1047, 1050, or 1062 nucleotides. However, three of these sequences displayed stop codons at the 29 (2 sequences) and 32 (1 sequence) amino acid coding sites. As a result, 23 alleles without premature stop codons could be translated successfully to proteins. According to comparisons with HLA amino acid sequences (GenBank accession number: AAA76608.2), the four pivotal amino acid sites that form disulfide bridges (Cys203-Cys259 and Cys101-Cys164) were conserved in the 23 sequences (Figure 2).

**Figure 3:**
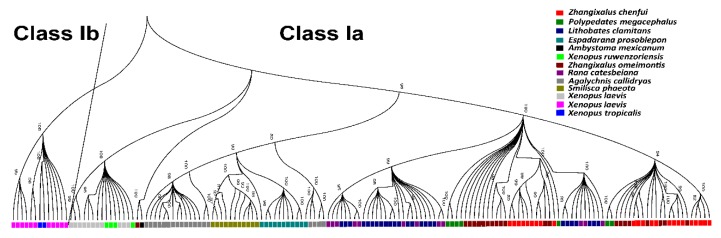
Phylogenetic relationships of anuran *MHC class Ia* and *Ib* genes. The neighbor-joining tree was constructed with MEGA5 using all available anuran *MHC class I* genes with an available length. Bootstrap values are indicated above the branches. Sample names are not shown due to the large size of the tree. Rather, the species to which these samples belong are denoted with different colors. The horizontal line separates *MHC class Ia* genes (to the right of the line) and *MHC class Ib* genes (to the left of the line).

This suggested that these isolated alleles may be functional. It was further shown in the phylogenetic analyses that the 23 alleles were placed at the *MHC class Ia* locus (Figure 3). Alleles were submitted with accession numbers (KX467504-KX467526) to GenBank.

### 3.3. Number of Loci and Genetic Diversity

In this study, we identified 2 alleles in 3 individuals, 3 alleles in 4 individuals, 4 alleles in 4 individuals, 5 alleles in 1 individual, and 6 alleles in 1 individual. At most, six alleles were observed in each Zc*MHC class Ia* gene. Since Chenfu’s Treefrog is diploid [33], we hypothesized that it possesses 2 to 3 loci for *MHC class Ia*. Additionally, phylogenetic analysis showed that all alleles were clustered into three branches (Figure 3). Therefore, Chenfu’s Treefrogs have at least three *MHC class Ia* loci.

Comparatively, high genetic diversity was observed in the sequences of *MHC class Ia* in Chenfu’s Treefrogs (Table 2). In Chenfu’s Treefrogs, the α3 domain had the lowest diversity whereas the α1 domain had the highest. Since the amino acid chain of the leading peptide, cytoplasmic region, and the trans-membrane region was shorter, there is a larger standard error in these regions. In addition, in comparison with the degree of divergence amongst amino acid chains, less divergence was observed amongst nucleotide sequences.

### 3.4. Recombination Detection

The recombination test was performed before the selection test, given that false inference of positive selection can be generated by recombination. The RDP4 program was used and three alleles were identified as products of recombination (Table 3). The actual number of recombinants is likely more than three as conservative parameters were used to avoid miscalculations. Nonetheless, these three alleles represented a marked proportion (13.04%) of the alleles. The RDP4 program was used to detect the five breakpoints (234, 314, 764, 993, and 1046).

### 3.5. Selection Detection

Since recombination mainly occurs at the boundary of different MHC domains, in order to avoid the influence of recombination on the selection signal, we divided *MHC class I* into six parts: leading peptide, α1 domain, α2 domain, α3 domain, transmembrane region, and cytoplasmic region. In order to screen for selection signals, three different codon-oriented maximal likelihood approaches were applied, FEL, PAML, and MEME, despite each approach identifying a different number of positively selected sites (Figure 2 and Table 4). In total, 21 sites of Chenfu’s Treefrog *MHC class Ia* alleles were detected as under positive selection, eight of which were identified by more than one program. Of the 21 sites, 13 were assigned to the α1 and α2 domains. In these domains, excluding two sites on domain α1 and four sites on domain α2, the seven remaining sites all belonged to the presumed ABS [5] (Figure 2). We also tested for positively selected sites in the leading peptide, α3 domain, transmembrane region, and cytoplasmic region. We observed no positively selected sites in the leading peptide, two positively selected sites in the α3 domain, two positively selected sites in the transmembrane region, and three positively selected sites in the cytoplasmic region.

### 3.6. Phylogenetic Analyses

The 23 Chenfu’s Treefrog alleles and previously published *MHC class I* genes with comparable coverage from amphibians (see Methods) were used to construct the phylogenetic tree and resolve the phylogenetic status of the new alleles. We found that genes of *MHC class Ia* and *MHC class Ib* clustered separately and that the cluster of *MHC class Ia* genes contained all Chenfu’s Treefrog alleles (Figure 3).

**Figure 4:**
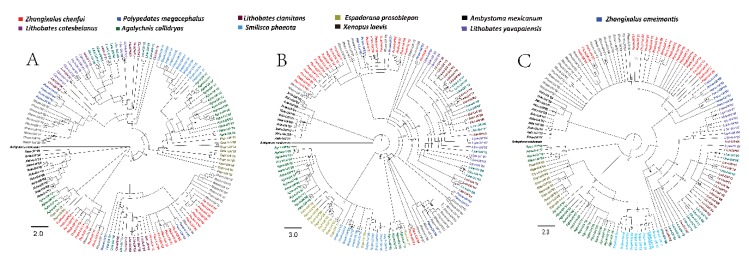
Phylogenetic relationships of α1 (**A**), α2 (**B**) and α3 (**C**) domains (analysis by MEGA). All of the branches are proportional to the scale shown at the bottom left of the figure. Alleles belonging to different species are marked using different colors.

Furthermore, to reconstruct a more accurate phylogenetic relationship, the aligned sequences were partitioned to individual domain regions, given that the exon exchanges took place between exons 3/4 and 2 and that mutation ratios were different between the α1, α2, and α3 domains. No specific topology of species was observed in the phylogenetic trees of the α1, α2, and α3 domains (Figure 4) when neighbor-joining (NJ) trees were constructed using synonymous mutations.

**Figure 5:**
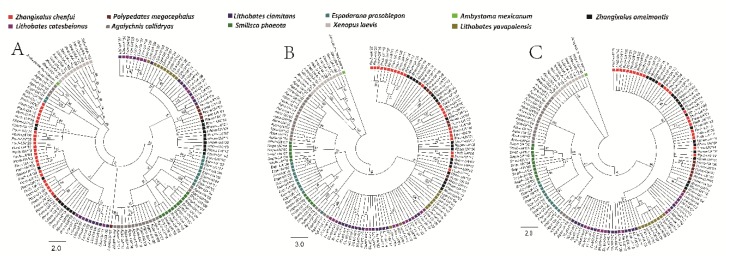
Phylogenetic relationships of α1 (**A**), α2 (**B**) and α3 (**C**) domains. Bayesian posterior probabilities are presented. All of the branches are proportional to the scale shown at the bottom left. Alleles belonging to different species are marked using different colored.

Trans-species polymorphisms of anuran *MHC class Ia* genes were evident considering that *MHC class Ia* alleles shared common ancestry (Figure 3). Alleles from different taxa in the phylogenetic trees of the α1, α2, and α3 domains were no less similar to each other than the alleles from the same species. A part of the Rhacophoridae alleles in the tree of α1 domains branched from the common ancestors Centrolenidae, Hylidae, Ranidae, and Rhacophoridae species; the other portion was bifurcated into two clusters (Figure 4A and Figure 5A). Though the tree showed a star-like topology because of the extensive non-synonymous mutations, a mixture of alleles from various species in the tree of the α2 domains could be observed as well (Figure 4B and Figure 5B, Table 1). The tree constructed for the α3 domains conformed to the species tree as there were four clusters which were consistent with the anuran taxa: the cluster of Ranidae as the sister clade of Rhacophoridae was formed by alleles from *Lithobates clamitans*, *Lithobates yavapaiensis*, and *Lithobates catesbeianus*; the Rhacophoridae cluster with *Zhangixalus omeimontis* and *Polypedates megacephalus* was formed by the newly obtained alleles from Chenfu’s Treefrog; a well-supported clade was formed by alleles from Xenopus; alleles from *Agalychnis callidryas* and *Smilisca phaeota* belonged to the Hylidae cluster and combined with alleles from *Espadarana prosoblepon* (Figure 4C and Figure 5C). However, a portion of the *Agalychnis callidryas* alleles formed an independent branch. In conclusion, trans-species polymorphisms were observed in α1 and α2 domains of *MHC class I* genes, and species-specific clustering was found in α3 domain of *MHC class I* genes.

## 4. Discussion

Full-length cDNA of *MHC class I* genes was isolated successfully from Chenfu’s Treefrogs. Afterward, we obtained 23 alleles from 13 individuals with full-length ORFs. Phylogenetic analyses suggested that all of the newly isolated alleles were contained within the *MHC class Ia* loci. Subsequent analysis showed that: (1) alleles clustered into three branches in the phylogenetic analyses and displayed a comparatively high genetic diversity; (2) the putative amino acids of all the alleles included those forming disulfide bonds; (3) three alleles were the products of recombination; and (4) the alleles showed positive selection on many putative antigen binding sites (ABS).

As far as we know, previous research focusing on the MHC of anurans was mainly focused on α1, α2, and α3 domains [31,32]. Therefore, this study has provided a more comprehensive information regarding the alleles of a species in the order Anura (Chenfu’s Treefrog). This enabled some interesting features to be discovered in the transmembrane and cytoplasmic regions. First of all, we found two positively selected sites in the transmembrane region and three positively selected sites in the cytoplasmic region. Second, we found breakpoints in the boundaries of both the transmembrane and cytoplasmic regions. Third, we located a potential N-linked glycosylation site in the cytoplasmic region of alleles. These findings suggest that further studies should focus on these areas. Furthermore, we observed N-glycosylation in the α1 region, which is important for assembling α-chains and transporting molecules of *MHC class I* through the Golgi apparatus and endoplasmic reticulum (ER) [35].

A great number of vertebrates have been observed to have high variation in MHC loci numbers, including fish [9,36], reptiles [37], birds [2,10,26], and mammals [15], which usually have more than one locus. For instance, three loci of *MHC class I* are expressed in Atlantic salmon [38], at least six loci are expressed in Blue tits [10], and 17 loci in a Cichlid species [39] but only two loci in Chinese sturgeon and paddlefish [9], as well as chickens [40]. According to prior research, a simple yet similar pattern for amphibians has shown that only one locus is usually expressed in Anura [30,41,42,43,44], whereas more than one locus is expressed in Caudata [27]. Researchers with less or insufficient information predicted that the diversification of amphibian loci of *MHC class I* took place sometime after the divergence of the Anura/Caudata but before that of the Pipidae/Ranidae [41] and that the number of loci of *MHC class I* was different amongst the anuran species [31,32].

We concluded that there are three *MHC class Ia* loci in Chenfu’s Treefrog, given that six alleles were apparent in some Chenfu’s Treefrog individuals included in this study and the knowledge that this species is diploid [33]. Due to our stringent criterion of allele identification, a large amount of sequence data (37 sequences) was discarded. Because alleles with low frequencies were removed, the actual number of loci of *MHC class Ia* in Chenfu’s Treefrog may have been underestimated. It is notable that more than three loci of *MHC class Ia* were observed in Chenfu’s frog. Prior research has shown that the Anura, including two Ranidae species (*Rana temporaria* and *Lithobates pipiens*) [41,42,43] and two Pipidae species (*Xenopus tropicalis and Xenopus laevis*) [30,44], expressed only one *MHC class Ia* locus, making three *MHC class Ia* loci quite uncommon in anuran species. However, according to recent studies on the evolution of the anuran MHC, non-model anuran species exhibited 2 or 3 for *MHC class Ia* loci [31,32]. In addition, a recent study showed that there are at least four loci in *Bufo gargarizans* and *Hyla japonica* [45]. Our study adds an additional anuran species with 3 *MHC class Ia* loci, which supports the ubiquity of multiple loci in the Auran *MHC class Ia*.

**Figure 6:**
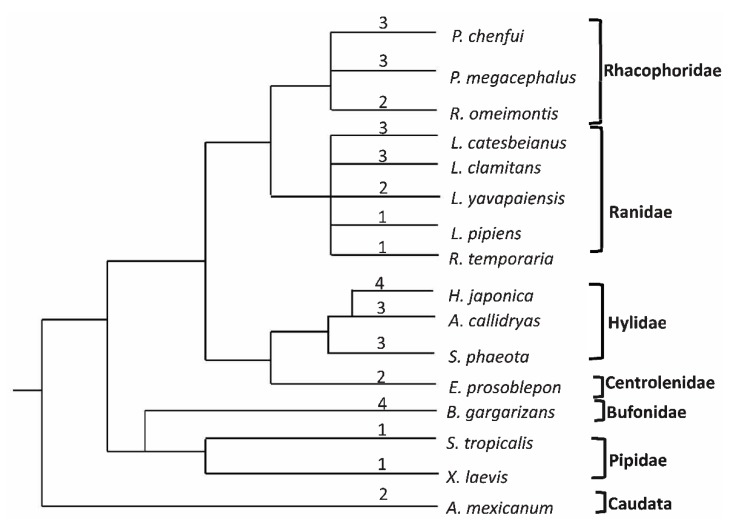
Numbers of putative *MHC class Ia* loci in amphibian species. The simplified phylogenetic tree of amphibian species with a deduced number of *MHC class Ia* loci based on the amphibian pedigree shown in Didinger et al., Zhao et al., and Roelants et al. The putative number of loci is shown above the corresponding branches.

The variation in number of amphibian *MHC class I* loci appears to be more complex. After all available information on amphibian *MHC class I* gene loci was combined, it was found that species in the same family can have different MHC loci numbers (Figure 6) [46]. This supports that the variation in loci number in anurans is species-specific. During the evolution of amphibian *MHC class I* genes, which is the birth-and-death model of evolution, the occurrence of gene loss/duplication can be used to explain the pattern of mixed loci numbers [47]. However, inaccurate estimations of loci numbers in every species can also contribute to this pattern, as the two causes are compatible. Furthermore, previous studies have reported differences in number of MHC loci amongst populations or individuals of the same species [16,31,48]. Therefore, it is too early to draw conclusions regarding these variations until further studies are conducted to preclude the possibility that the number of loci was underestimated. We propose that future studies should be conducted to establish accurate numbers for anuran *MHC class Ia* gene loci in order to understand its history of duplication. In addition, availability of whole genome sequence would also help with elucidating MHC copy number.

Recombination is one of the most significant drivers of high diversity of *MHC class I* genes and generation of novel alleles [7,8,9,10]. Here, recombination was found to play a significant role in the evolution of *MHC class Ia* genes of Chenfu’s Treefrog. Recombination generated 13.04% of the alleles. Furthermore, a large portion of the recombination breakpoints was found near the regional boundary, and the other portions were located near the two insertion mutation sites. It was found that the DNA was exchanged primarily between domains α1, α2, and α3. The recombination pattern is similar to that observed in the genes of *MHC class I* in bony fish and other anurans [8,9,28,49]. By looking at the pattern of exon shuffling, *MHC class I* genes of teleost and anuran species may share similar organizations. One previous study reported that intron 2 of *MHC class I* gene in bony fish contained numerous repeated elements and had long coverage [50]. These two gene organization forces played an important role in making intron 2 sequence recombination hot spots. Nonetheless, the intron 2 sequences of Rhacophoridae genes of *MHC class I* was not available for study. According to the genetic information from the entire genome of *Xenopus tropicalis*, it appears that Rhacophoridae species tend to have a long intron 2 regions of the *MHC class I* genes [28]. However, recombination analysis revealed breakpoints located near the two insertion mutation sites, which suggests that there are more complicated mechanisms and patterns involved in the recombination of Chenfu’s Treefrog genes of *MHC class I*. This indicates that the recombinational pattern in Chenfu’s Treefrog is similar to the mammals which show reticulate recombination patterns [51]. To gain more systematic and comprehensive information regarding recombination in amphibian *MHC class I* genes, a larger number of amphibian species should be evaluated.

In this study, using various approaches, it has been shown that Chenfu’s Treefrog *MHC class I* genes have experienced positive selection. With positive selection occurring in the 23 alleles of *MHC class I*, positive selection signals and sites were detected by multiple codon-oriented approaches. The sites, identified by more than one approach, were regarded robust and made up the subset of putative ABS from the human HLA crystal structure [5]. This result indicated that, despite some differences, the Chenfu’s Treefrog *MHC class I* molecules and human HLA may share some similarity in three-dimensional structure. Similar results have been observed in many other species [9,31,52]. It can be concluded that the genes of *MHC class I* genes tend to undergo pathogen-mediated positive selection due to their ABS function.

Given that there is common ancestry in the alleles of *MHC class Ia* from Rhacophoridae, Ranidae, Centrolenidae, and Hylidae (Figure 1), trans-species polymorphisms of anuran genes of *MHC class Ia* were evident. Specifically, the α1 and α2 domains of anuran *MHC class I* genes are where the trans-species polymorphisms were observed (Figure 5). The species-specific cluster was not observed in the phylogenetic trees of α1 and α2 (Figure 5) when NJ trees were constructed using synonymous mutations. Later, positive selection was identified to match the pattern of trans-species polymorphism. To be more specific, α1 and α2 containing major regions for antigen binding and recognition will experience a strong positive selection force. By encoding the domains, nucleotide sequences can be retained for long periods of time and the origin of alleles with sufficient importance predates the divergence of species. On the contrary, α3 functions as a link and an anchor and experiences relatively little selection pressure. Consequently, a precise phylogenetic relationship of these species can be established by examining the commonality in the α3 domain. Previous studies have also reported differences in patterns of evolution for different exons of the same gene [9,32,53].

The mixture of alleles present in various species as trans-species polymorphisms speaks to the long history of those genes in the MHC, predating the emergence of those species [54]. Therefore, the time of MHC gene formation can be inferred from the divergence time of related species. Before the divergences of Centrolenidae/Hylidae and Rhacophoridae/Ranidae (Figure 5A), based on this research, the allelic lineages of *MHC class Ia* found in Chenfu’s Treefrog already existed. The oldest allelic lineages of *MHC class Ia* in Anura have been predicted to have originated around 70 MYA (million years ago) as the two divergence events date back to 68–91 and 69–72 MYA [55]. The time that the alleles of *MHC class I* genes have been persisted in Anura is longer than that of certain other vertebrates [56].

## 5. Conclusions

In summary, *MHC class Ia* genes were isolated successfully from *Chenfu’s Treefrog*, which were found to contain three loci for *MHC class Ia* genes. Because of exchanges of entire exons and positive selection acting predominantly on the ABS, frequent gene duplication and recombination played significant roles in the evolution of the genes of Rhacophoridae *MHC class I*. Our knowledge of the evolution of the anuran *MHC* genes has been expanded upon by the inclusion of Rhacophoridae *MHC class I* genes.

## Figures and Tables

**Table 1:** Primer sequences and function used in this study.

Primer Name	Primer Sequence (5-3′)	Applications
ZcMHC-F	5′CTGCGSWAYTATKABACWGCAGTCTC 3′	ZcMHC cloning
ZcMHC-R	5′TYCAGRCTGCTGTGSTCCACAT 3′	ZcMHC cloning
ZcMHC-F1	5′CCGTCAGTCCGATGGTGCGA 3′	ZcMHC 3′RACE outer
ZcMHC-F2	5′GAAGTGCCAACAACCGAAGGAGAC 3′	ZcMHC 3′RACE inner
ZcMHC-R1	5′GAGCCTCGTTCATGGTGGGGATA 3′	ZcMHC 5′RACE outer
ZcMHC-R2	5′GAGTCACAGGACGAGCCTGATGA T3′	ZcMHC 5′RACE inner
ZcMHC-F3	5′GAGGTGACACACAGGAGGAGATGGA 3′	ZcMHC coding region PCR
ZcMHC-R3	5′CGGCATRGTGTACGCTCAGGCTKT 3′	ZcMHC coding region PCR

Primers were designed using primer premier 5.0 (PREMIER Biosoft International, Palo Alto, CA).

**Table 2:** Average nucleotide and amino acid distances among the 23 newly isolated alleles.

Amino Acid.	K2P Nucleotide Distance	Standard Error	Poisson-Corrected Amino Acid Distance	Standard Error
complete sequence	0.075	0.005	0.130	0.013
leading peptide	0.014	0.007	0.042	0.021
α1 domain	0.170	0.020	0.295	0.040
α2 domain	0.089	0.012	0.163	0.030
α3 domain	0.014	0.003	0.030	0.009
transmembrane region	0.062	0.020	0.144	0.059
cytoplasmic region	0.062	0.018	0.126	0.045

**Table 3:** Recombination test using the RDP4 program.

Recombinant Sequence	Nucleotide Breakpoint	Potential Parental Sequences	Methods
Poch-UA*05	234,764	Poch-UA*07/Poch-UA*18	RDP, GENECONV, BootScan, MaxChi, Chimaera, Siscan, 3Seq
Poch-UA*09	314,1046	Unknown(Poch-UA*14)/Poch-UA*11	GENECONV, BootScan, MaxChi, Chimaera, Siscan, 3Seq
Poch-UA*18	234,993	Unknown(Poch-UA*07)/Poch-UA*10	BootScan, MaxChi, Chimaera, Siscan, 3Seq

Notes: Unknown was used to indicate an indeterminate source of reorganization.

**Table 4:** Summary statistics for codon sites undergoing positive selection identified by different methods.

Method	α1 Domain				α2 Domain										α3 Domain		Transmembrane Region		Cytoplasmic Region		
	11	42	80	83	7	23	25	34	37	42	63	67	74	84	85	99	5	9	1	17	23
**FEL** ^**a**^		+	+					+			+	+						+		+	
**MEME** ^**b**^	+	+	+	+		+		+	+		+	+	+	+			+		+	+	
**Codeml** ^**c**^		*			**	*	**	*		**	**	**			**	**			*		*

Notes: Numbers correspond to the alignment shown in Figure 2. ^a^ Amino acid sites identified by FEL are identified with +. ^b^ Amino acid sites identified by MEME are identified with +. ^c^ Amino acids were identified in model M8 by the Bayes empirical Bayes procedure using Codeml. Only sites that were predicted to have undergone positive selection with a posterior probability >95% are presented in the table. * indicates a posterior probability is >95%, and ** indicates posterior probability is >99%.
